# Metabolic regulation boosts bioelectricity generation in *Zymomonas mobilis* microbial fuel cell, surpassing ethanol production

**DOI:** 10.1038/s41598-023-47846-7

**Published:** 2023-11-24

**Authors:** Hananeh Ahmadpanah, Ehsan Motamedian, Mohammad Mahdi Mardanpour

**Affiliations:** 1 Department of Biotechnology, Faculty of Chemical Engineering, Tarbiat Modares University, P.O. Box 14115‑143, Tehran, Iran; 2 Department of Bioengineering, Faculty of Engineering, McGill University, Montreal, Canada

**Keywords:** Biological techniques, Biotechnology

## Abstract

*Zymomonas mobilis* (*Z. mobilis*), a bacterium known for its ethanol production capabilities, can also generate electricity by transitioning from ethanol production to electron generation. The purpose of this study is to investigate the ability of *Z. mobilis* to produce bioelectricity when utilized as a biocatalyst in a single-chamber microbial fuel cell (MFC). Given the bacterium's strong inclination towards ethanol production, a metabolic engineering strategy was devised to identify key reactions responsible for redirecting electrons from ethanol towards electricity generation. To evaluate the electroactivity of cultured *Z. mobilis* and its ethanol production in the presence of regulators, the reduction of soluble Fe(III) was utilized. Among the regulators tested, CuCl_2_ demonstrated superior effectiveness. Consequently, the MFC was employed to analyze the electrochemical properties of *Z. mobilis* using both a minimal and modified medium. By modifying the bacterial medium, the maximum current and power density of the MFC fed with *Z. mobilis* increased by more than 5.8- and sixfold, respectively, compared to the minimal medium. These findings highlight the significant impact of metabolic redirection in enhancing the performance of MFCs. Furthermore, they establish *Z. mobilis* as an active electrogenesis microorganism capable of power generation in MFCs.

## Introduction

The capability of electrochemically active microbes to extract the electrons in organic waste and transfer them extracellularly exceedingly attract academic interest^1–3^. The application of these microorganisms in bioenergy production (in the forms of hydrogen^4^, biofuel^5^, or bioelectricity^6^) and developing a new approach to treat organic wastewater are significant potentials that have turned this issue into one of the hot academic topics^7^. The microbial fuel cell (MFC) is an innovative platform to recruit electrochemical active microorganisms for bioelectricity production^8,9^. Although the MFCs’ applications in power generation and waste treatment^10–13^ were demonstrated, the chief barrier to accelerating the commercialization of MFCs is their low performance^14,15^. It is obvious that the activity of electrogenesis microorganisms plays the most critical role in this issue^16,17^, and manipulating metabolic reactions could be a unique and influential strategy to improve the efficiency of MFCs^18–20^.

The redox potentials of the involved species^21,22^, the microbial oxidative metabolism^23^, and the extracellular electron transfer (EET) mechanism^20,24^ affect the rate of electron production in MFCs. Considering the metabolic reactions of electrochemically active microorganisms, an increase in the concentration of Nicotinamide adenine dinucleotide (NAD) + hydrogen (H) (namely, NADH) improves the efficiency of EET^25^. Li et al. proved that metabolic engineering methods could be employed to increase the intracellular ratio of NADH/ NAD^+^, and consequently electron generation^26^. The cofactors NADH and NAD^+^ are essential for microorganism metabolism and their balance as a prerequisite for higher electricity output is crucial.

*Zymomonas mobilis* (*Z. mobilis*) is an ethanologenic bacterium that keeps redox balance during ethanol fermentation in the glycolysis pathway^27,28^. This bacterium metabolizes sugar via the Entner–Doudoroff (ED) pathway in conjunction with the enzymes pyruvate decarboxylase and alcohol dehydrogenase to produce ethanol. ED is an alternative glycolysis pathway, and in this pathway, glucose is converted directly to pyruvate without the production of adenosine triphosphate (ATP) or indirectly changed to pyruvate based on ATP production^29^. The fermentation pathway of *Z. mobilis* for ethanol production has a relatively simple respiratory system and it is the main pathway for electron transport. It chiefly consists of a type II NADH dehydrogenase (NDH), coenzyme Q10, and cytochrome bd terminal oxidase, and cytochrome C peroxidase as the major electron carriers^30^. One of the main consumptions of NADH in *Z. mobilis* catabolism is ethanol fermentation^31^. NADH is used as an electron carrier by respiratory chain NADH dehydrogenase, and NADH is oxidized by alcohol dehydrogenase of the pyruvate-to-ethanol pathway in ethanologenic *Z. mobilis*. The TCA cycle in bacteria is incomplete and does not function as catabolic pathways. Therefore, NADH can only be synthesized from GAP and glucose-6-phosphate dehydrogenase (ZWF) in the ED pathway^32,33^. The EET pathway transports intracellular electrons from NADH to extracellular electron acceptors^25^. Regarding the ability of *Z. mobilis* to transfer electrons extracellularly^20^, Yu-Geng et al. illustrated that *Z. mobilis* was able to generate considerable bioelectricity (2.0 mW/m^2^). In this research, to improve the microbial fuel cells several strategies were employed which most noticeable of them were the removal of biofilm and using Tween 80. The removal of the biofilm and c-type cytochrome contributed to the bioelectricity production of the cell, and also by using Tween 80 a higher voltage output was achieved. This revealed that the redox balance is an important requirement for higher electricity output^34^. But so far, the performance of microbial fuel cells inoculated with *Z. mobilis* bacteria has not been studied and the technique of metabolic engineering to improve its efficiency has not been investigated.

Regarding the performance of *Z. mobilis,* some studies used metabolic engineering strategies to produce byproducts by reducing the efficiency of ethanol production. Oliver et al.^35^ investigated the active respiratory chain of *Z. mobilis* to regenerate NAD+ and produce acetaldehyde by developing the stoichiometric model of the central metabolism. In their work, *Z. mobilis'* electron transport chain was demonstrated to be capable of balancing the redox state. By increasing flux through the electron transport chain and reducing the activity of the alcohol dehydrogenase reaction instead of ethanol, the production of acetaldehyde increased and the yield of acetaldehyde would reach 70% of the theoretical maximum value. Also, in another research, Yang et al.^27^ improved the yield of bioproducts such as lactate and isobutanol by the redirection of central carbon metabolism in *Z. mobilis* to redox-balanced products. *Zymomonas mobilis* pdc under inducible diverts pyruvate accumulation to other pathways as ethanol production is inhibited in PDC depletion conditions. Anaerobically, Lactate and isobutanol were produced at high yields from glucose using the PDC inducible strain. Lactate yield reached 70% and isobutanol yield reached 65% of theoretical maximums, respectively. So, these reports demonstrate that *Z. mobilis* has the potential of redirecting electron from ethanol production to electricity generation which has not been studied.

Regarding the issue that ethanol production in *Z. mobilis* aims to balance NADH/NAD^+^ on the one hand and electron balance in the cell on the other hand^27^, *Z. mobilis* has a high potential for electron production. In this study, a metabolic engineering strategy was utilized to design a regulatory-defined medium and influence the metabolic flux in *Z. mobilis* in order to shift NADH from ethanol production to electron production. This causes an increase in the regeneration of intracellular NADH which increases the NADH/NAD^+^ ratio and consequently can increase electron generation. To achieve this, a framework for identifying critical reactions that enhance electricity production in *Z. mobilis* was comprehensively investigated by useful technique flux balance analysis (FBA) as a constrained-based modeling approach to find multiple optimal solutions^18^. The model was capable to identify up-regulated and down-regulated reactions by comparing the fluxes of each reaction under NADH and ethanol maximization in order to redirect electron to produce more instead of ethanol production. In the second step, the enzyme regulators found in BRENDA^36^ were added to the culture medium to regulate target reactions. Finally, this modified medium was used to inoculate an MFC and the performance of the MFC was evaluated by comparing polarization and power density curves under various conditions.

## Material and methods

### In silico simulation

To find the desirable metabolic reactions and the related compounds, in-silico analysis using iEM439, the GEM for *Zymomonas mobilis* ZM1 was performed. This model includes 439 genes, 692 metabolic reactions, and 658 metabolites^37^. The distribution of metabolic flux was modeled using flux balance analysis (FBA), a constraint-based modeling approach. In this analysis, it is assumed that an organism will reach a steady state under any given environmental condition^38^. A pseudo reaction for reduction of NAD to NADH was added to the metabolic model and considered as a objective function to calculate the maximum possible rate of electrons production. All reversible reactions were set to 1000 and − 1000 mmolgDCW^−1^ h^−1^, except the ATPS4rpp reaction, whose bound was set to − 4 mmolgDCW^−1^ h^−1^^37^. The intercellular irreversible reaction fluxes ranged between 0 and 1000 mmolgDCW^−1^ h^−1^ while the range for reversible reactions was between − 1000 and 1000 mmolgDCW^−1^ h^−1^. In order to simulate the growth of *Z. mobilis* in a glucose minimal medium, the lower bound of all exchange reactions except glucose, NH_4_^+^, H_2_O, SO_4_^−2^, O_2_, H^+^, PO_4_^−2^, and inorganic ions were set to zero. In addition, under anaerobic conditions, the lower bound for the oxygen exchange reaction was set to 0 mmolgDCW^−1^ h^−1^^37^. All in-silico and batch-culture simulations were run with the glucose uptake rate setting at − 10 mmolgDCW^−1^ h^−1^. The simulation was done with MATLAB (R2017b) software and COBRA Toolbox and GLPK (GNU Linear Programming Kit) were used to solve linear programming problems^39^.

### Identifying effective reactions and designing the regulatory defined medium

The LAMOS^18^ algorithm was applied to identify flux variability of each reaction and compare the metabolic reactions causing NADH and ethanol synthesis. By setting an objective function (generation of NADH or ethanol), FBA determined the maximum value of the objective function as the optimal viable solution by linear programming (LP)^40,41^. For all in-silico experiments, initially, the cell growth of the strain was simulated in the glucose minimal media, and the maximum biomass generating reaction was defined as an objective function. The optimal growth rate was set as a constraint in the following steps of simulation. Then, by limiting 100% of the optimal growth rate and setting the NADH or ethanol synthesis reaction in the metabolic network as the second objective function, the maximum NADH or ethanol production rate was obtained, and hence, the optima; value was bound in the metabolic model. Ultimately, for both ethanol and NADH objective functions, the model computes 10,000 optimal solutions. Using a threshold of 10^−8^, active reactions were identified, and then, for each of these conditions of maximum NADH and ethanol production, the flux variability for each reaction was calculated.

**Figure 1:**
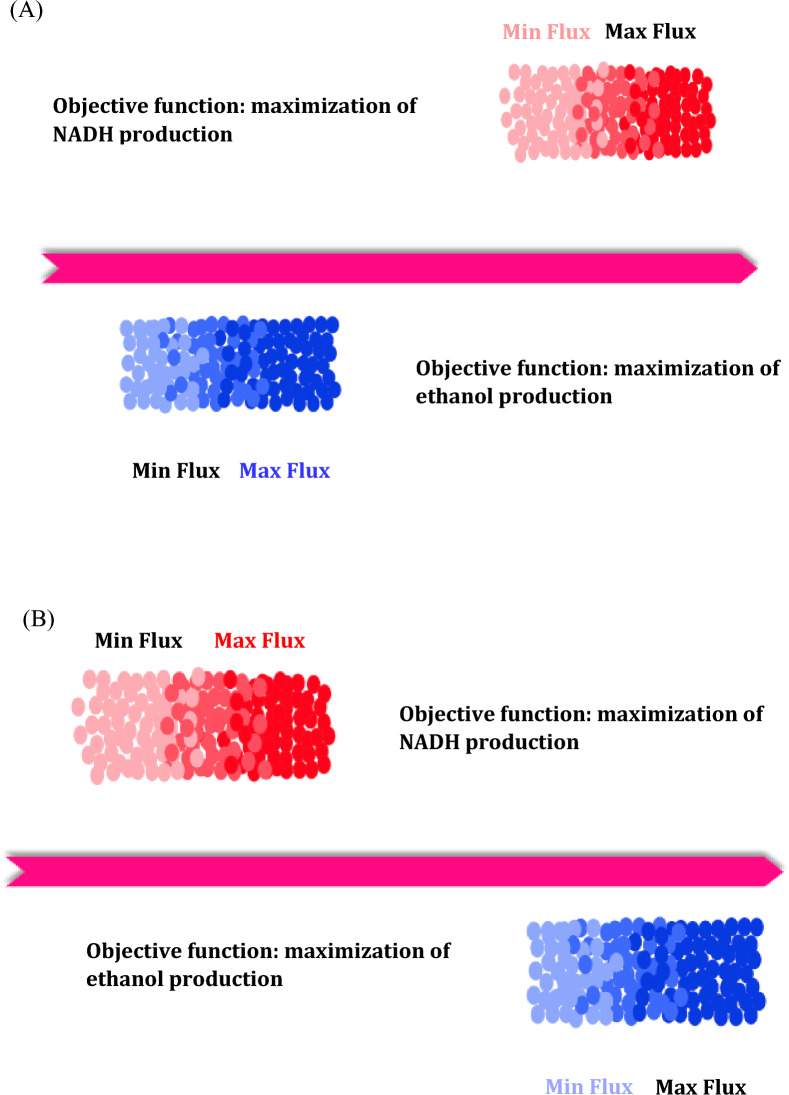
Comparison of flux variability for each reaction at the maximum rates of NADH and ethanol production reaction to finding appropriate reactions for (**A**) up-regulation, and (**B**) down-regulation.

In order to identify the candidate reactions for up or down regulations, two criteria were considered. The first criterion, as demonstrated in Fig. 1, is that a reaction is appropriate for up-regulation if its minimum flux in the maximum NADH production rate is higher than its maximum flux in the maximum ethanol production rate. In contrast, a metabolic reaction is proper to be down-regulated if its flux under the ethanol maximum flux is higher than its flux under the NADH maximum flux. The second criterion was based on the activity difference for each metabolic reaction. It was calculated as a fraction of the entire solution (10,000 optimal solutions) where the reaction was classified as an active one in the first criterion. By comparing desirable and undesirable conditions, a positive difference indicated an up-regulation of a metabolic reaction and a negative difference implied a down-regulation of the reaction. Based on the simulation results, the target enzymes were identified, and by searching in the BRENDA database, the regulatory compounds that control the activity of the target enzyme were extracted. Following that, to assess the capability of *Z. mobilis* in electron generation by metabolic engineering, these regulators were added to the medium culture, and the effects of up- or down-regulation of predicted reactions were investigated.

### Microorganism and culture media

*Z. mobilis* (ATCC 10,988, PTCC 1718) was taken from the Persian Type Culture Collection. The strain was grown in 50 ml of a complex medium containing 10 g L^−1^ peptone from meat, 10 g L^−1^ yeast extract, and 20 g L^−1^ glucose and cultured in a shaker incubator at 30 °C, 150 rpm for 14 h. Then, the culture approached medium-logarithm-phase conditions and it inoculated in the minimal medium. The composition of the minimal medium was glucose 20 g L^−1^, KH_2_PO_4_ 1 g L^−1^, K_2_HPO_4_ 1 g L^−1^, (NH_4_)_2_SO_4_ 1 g L^−1^, NaCl 0.5 g L^−1^, MgSO_4_·7H_2_O 0.2 g L^−1^, CaCl_2_·2H_2_O 0.2 g L^−1^, Na_2_MoO_4_·2H_2_O 0.025 g L^−1^, FeSO_4_·7H_2_O 0.025 g L^−1^^37^. After 18 h, the strain cultured in the minimal medium can be used to inoculate the MFC.

### Analytical method to screening compounds

By using the BRENDA database and identifying the effective reactions through simulation and the LAMOS algorithm, the substances restricting the reactions of ethanol production were identified. In order to increase extracellular electron transfer, the additive must first decrease ethanol production and then increase the production of an electron. So, to choose the most effective substance, gas chromatography GC-2550TG (column temperature 150 °C, injector temperature 250 °C, detector temperature 250 °C, and FID detector type (Tief Gostar Faraz Company, Iran)) was used to measure the ethanol concentration and ferrozine iron kits (Zist Chem Diaqnosis, Iran) as a method to examine the amount of the electron generated, were applied. The amount of biomass was measured using a spectrophotometer (Cary50Conc, UV–vis Spectrophotometer, Australia) at the same time interval and an optical density of 600 nm. The remaining glucose in the culture medium was quantified using a colorimetric glucose oxidase kit (Pars Azmun, Iran). It was an enzymatic method based on glucose oxidation by glucose oxidase enzyme.

#### Iron reduction assay

The electroactivity of *Z. mobilis* was investigated using a soluble Fe (III) reduction method. It is commonly used for detecting microbial EET activity and can be measured using the ferrozine complex, a colorimetric dye^42^. This complex is a bacteria-produced Fe (II) and the intensity of the light absorbed at 560–600 nm was used to measure Fe (II) concentration. The minimal medium with inhibitor concentrations was inoculated with a 10% inoculum of the middle logarithmic phase. After growing the strain for 12 h in any new culture, a ferrozine reagent was used to determine Fe (II) concentrations in each sample. In addition, based on Fe (III) reduction assays, for the minimal medium without inhibitors, Fe (II) concentrations were measured for 12 h, and the results were analyzed to determine appropriate materials for modification of the minimal medium.

### MFC assembly

The single chamber air cathode (MFC) with a total volume of 18 cm^3^, an electrode surface area of 9 cm^2^, and a height of 2 cm was designed and fabricated. This device is composed of three two cubic (Poly methyl methacrylate) (PMMA) chambers (1 cm in height, 7 cm in width, and 7 cm length), and the center of these chambers is a square with the size of 3 × 3 cm was cut using a laser beam to place anode and cathode electrodes.

**Figure 2:**
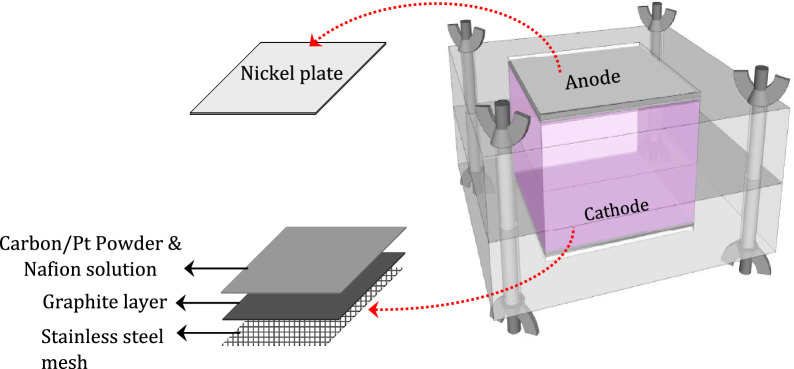
The schematic detail of the fabricated MFC.

The selected materials for MFCs’ electrodes should be biocompatible, corrosion-resistant, with high surface area, low electrical resistance, and chemically stable^43–45^. Anode characteristics affect biofilm formation, electron transfer, and substrate oxidation, making its material a crucial component in the performance of MFCs^46^. Nickel electrodes were found to provide a higher power density than carbon cloth^47^. Due to the hydrophilic nature of nickel, it is capable of rapidly absorbing anolyte and attaching biocatalysts instantly, which drastically increased the bioelectricity generation. Therefore, nickel was selected for the anode electrode. The anodic compartment was covered with a nickel plate (0.07 mm thick, 4 cm in width, and 4 cm in length). Regarding the importance of the surface area to expedite the cathodic reactions, graphite-coated stainless-steel mesh (mesh 400) was used for the cathode of the MFC. It was treated to achieve 0.5 mg cm^−2^ platinum (Pt) loading according to the previously published study^48^. The presence of Pt plays a critical role in the reduction of the activation over potential^49^. Bolts and nuts were inserted into holes at four corners of the top and bottom chambers. A schematic of the fabricated MFC is shown in Fig. 2.

### Microbial enrichment of MFC

Considering the effective growth of biofilm under the open circuit conditions^50^, the start-up of the MFC was done during the measurement of the open circuit potential (OCP). The MFC were fed glucose as a substrate, and the anolyte`s volume was refilled with a fresh medium whenever a huge reduction in the volume of the anolyte due to the substrate evaporation and bacterial activity was observed. The OCP of the MFC was monitored in two cases, it was first done for the MFC inoculated by the minimal medium and without addition of any regulatory substance and then it was fulfilled for the MFC inoculated with the minimal medium enriched by an appropriate regulatory substance. The selection process of an appropriate regulatory substance was explained in "The effect of the selective regulators on ethanol production of Z. mobilis" section.

The volumetric current density I_V_ (A m^3^) and coulombic efficiency (CE) of the MECs were calculated using the following equation in order to assess their performance^49^:

1 $${C}_{E}=\frac{{M}_{S}\underset{0}{\overset{{t}_{b}}{\int }}I\times dt}{F\times {b}_{es}\times v\times \Delta C}$$

where M_S_ indicates the molecular weight of the substrate (g/mole), t_b_ is time (s), F represents Farada’s constant (C/mole^−^), ν indicates the volume of anolyte (L), ∆C is the concentration difference during a defined period (g/L), b_es_ denotes the number of electrons exchanged per mole of oxygen (mole^−^/mole), and finally, I indicates the produced current (A).

## Results and discussion

### Analyzing the results of the algorithm LAMOS

**Figure 3:**
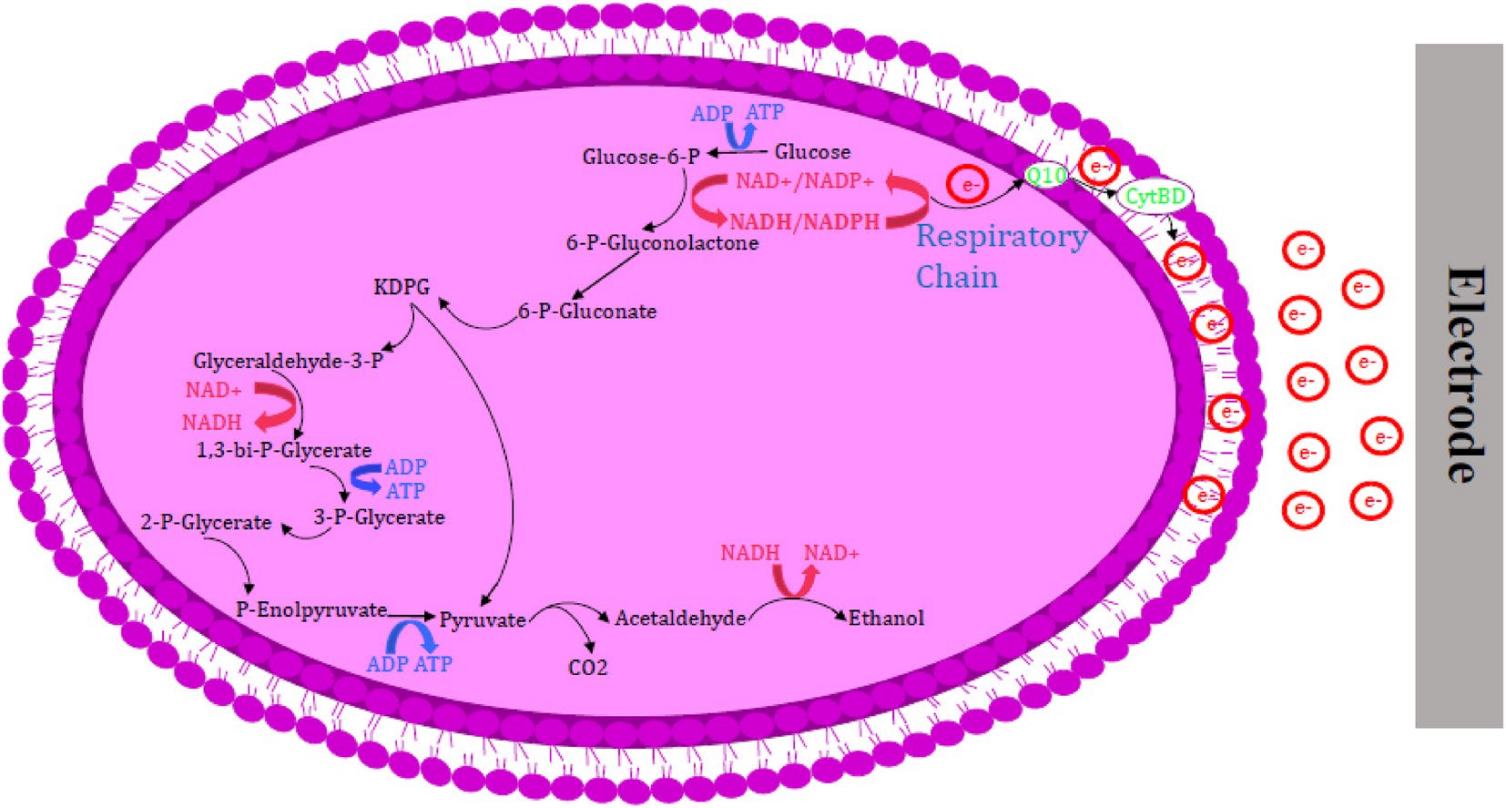
The overview of NADH production pathway. The reaction of ethanol production is a suitable candidate for down-regulation.

Based on the results of the simulation by the LAMOS algorithm, the effective reactions to increase NADH production were detected. The details of these reactions are presented in supplementary materials, Tables S1 and S2. Figure 3 illustrates the effective metabolic pathways on the overproduction of NADH and consequently the enhancement of electron generation. According to the LAMOS algorithm, the alcohol dehydrogenase enzyme responsible for reaction ALCD2x_b (converting acetaldehyde to ethanol) was selected to be inhibited. Due to the activity of the pyruvate decarboxylase (PDC) enzyme, pyruvate is converted to acetaldehyde and carbon dioxide during an irreversible reaction in *Z. mobilis.* Ethanol is naturally produced along with the reduction of acetaldehyde in the presence of alcohol dehydrogenase which its production results in ATP production. ATP is produced by NAD^+^ in the glycolysis cycle and it is necessary to generate NAD^+^ and maintain energy production. To produce NAD^+^, 3-phosphate glycerol (g3p) must be oxidized by an electron receptor during glycolysis reaction (GAPD-f reaction). Thus, the electron is transferred from NADH to acetaldehyde, and under the influence of the enzyme alcohol dehydrogenase, acetaldehyde is converted to ethanol (ALCD2x-f reaction)^29,51^. In this manner, by targeting the enzyme alcohol dehydrogenase and inactivating it, the ethanol production pathway is blocked, therefore, NADH consumption for ethanol is prevented and it is reserved for electron generation.

### The effect of the selective regulators on ethanol production of *Z. mobilis*

**Figure 4:**
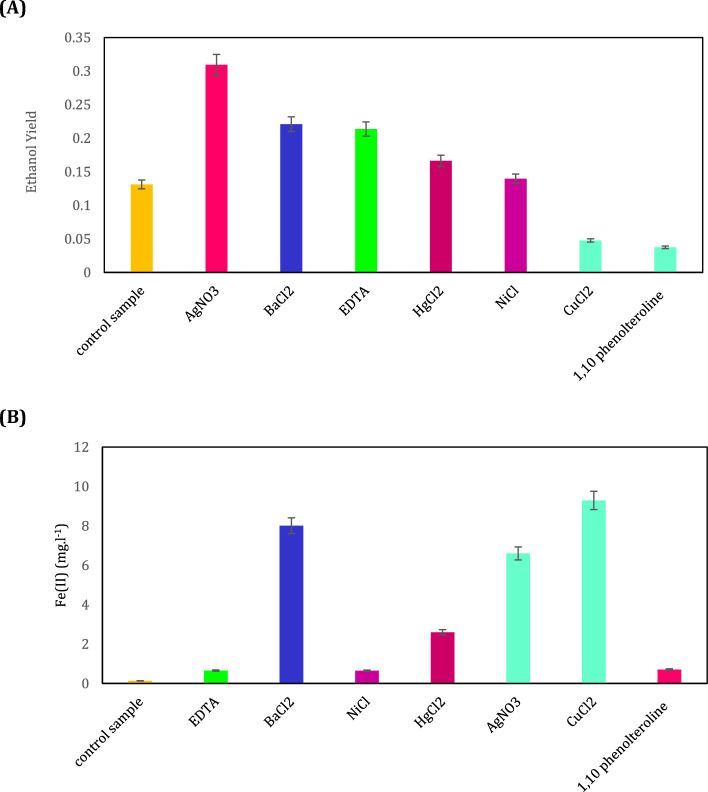
The effect of different regulatory compounds on (**A**) the amount of yield of ethanol production (namely the concentration of produced ethanol (g L^−1^) to the concentration of consumed glucose (g L^−1^)), and (**B**) the reduction of Fe (III) of the culture media of *Z. mobilis.*

The effect of different regulatory compounds on the yield of ethanol production (namely the amount of produced ethanol (g L^−1^) to the amount of consumed glucose (g L^−1^)) is shown in Fig. 4A. The first bar in this chart represents control sample namely *Z. mobilis* cultured in the minimal medium and other bars indicate the influence of the selected regulatory compounds on the yield of ethanol production by adding them to the *Z. mobilis* medium. The ethanol production of *Z. mobilis* in the medium without regulatory compounds was presented in the Supplementary file 1 (Fig. S1). A remarkable and gradual increase in ethanol production in the absence of the regulatory compounds was obvious in Fig. S1. This implied the significant role of the regulatory compound in changing the direction of ethanol production to electron generation.

As shown in Fig. 4A, by using AgNO_3_, BaCl_2_, EDTA, NiCl_2_, and HgCl_2_ the yield of ethanol compared to the control sample increased 135.98%, 68.46%, 62.93%, 6.47%, and 26.84%, respectively. On the other hand, the use of CuCl_2_ and 1, 10-phenanthroline decreased the yield of ethanol by 63.65% and 71.46%, respectively. This indicated that CuCl_2_ and 1, 10-phenanthroline were the most influential compounds of this stage. According to the BRENDA database, it was reported that 1 mM CuCl2 causes 68% inhibition of alcohol dehydrogenase of *Flavobacterium frigidimaris* bacteria^52^, and 0.2 mM 1,10-phenanthroline is a strong inhibitor for the ethanol production of *Z. mobilis*^53^.

### The effect of the selective regulators on the electron production of *Z. mobilis*

As mentioned in "Iron reduction assay" section, in order to analyze EET activity for *Z. mobilis*, the reduction tests of Fe (III) namely ferrozine assays were conducted. Regarding the reduction reaction of Fe (III) to Fe (II) (Fe (III) + e^−^ → Fe (II)), the higher concentration of Fe (II) in the mixture indicated a higher rate of electron production of inoculated bacteria. The amount of Fe (II) concentration is illustrated in Fig. 4B.

As can be seen in Fig. 4B, the EET activity of cultured *Z. mobilis* in the minimal medium was higher in the presence of BaCl_2_, AgNO_3_, and CuCl_2_ compared to other additives. In other words, these additives decrease ethanol production. The highest concentration of Fe (II) in the modified medium containing CuCl_2_ reveals that this substance is the most effective regulator to revolve the EET activity of *Z. mobilis*. To speculate the evolution of electron production of *Z. mobilis*, the reduction of Fe (III) in the cultured *Z. mobilis* without a regulatory compound as a control sample was also achieved and shown in the Supplementary file 1, Fig. S2. Therefore, during the experiments, the concentration of CuCl2 was set at 1 mM at the beginning of the culture medium. The effect of CuCl2 on reducing the activity of alcohol dehydrogenase enzyme in vitro by performing enzyme assay has been proven in the previously published study^52^. Therefore, the present work mainly focused on the implementation of the modified culture medium to motivate *Z. mobilis* towards electron production and evaluate its performance by the power generation platform.

### Monitoring open circuit potential (OCP)

Microbial enrichment was done during open circuit conditions. Two types of anolytes were used for this stage: (i) the cultured *Z. mobilis* in the minimal medium and (ii) the cultured *Z. mobilis* in the modified medium (namely the minimal medium with adding CuCl_2_ as selective regulator).

**Figure 5:**
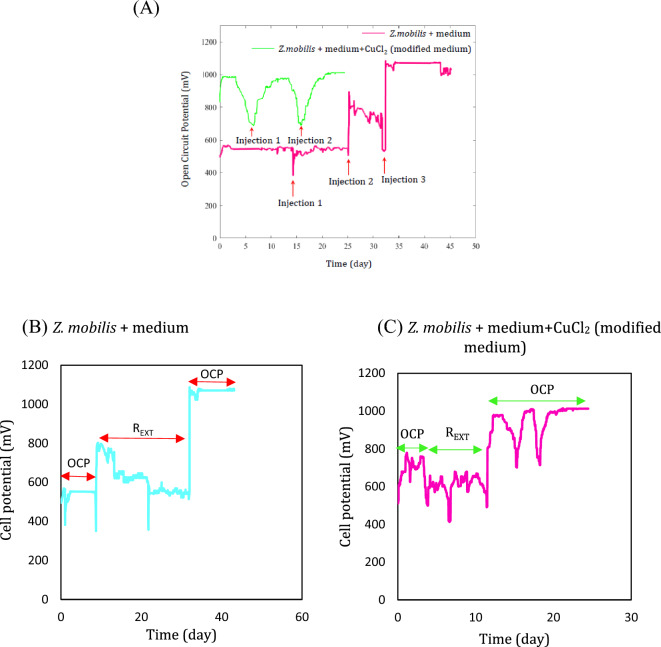
(**A**) The evolution of open circuit potential of two kinds of medium including minimal (medium + *Z. mobilis*) and modified (medium + *Z. mobilis* + CuCl_2_) mediums; The evolution of cell potential by changing open circuit to closed circuit conditions and applying an external resistance in two kinds of medium including (**B**) *Z. mobilis* cultured in the minimal medium (**C**) *Z. mobilis* cultured in the modified medium (medium + *Z. mobilis* + CuCl_2_).

To maximize the flux of extracted electrons from *Z. mobilis*, and reduce the electrical loss, the growth of an effective biofilm is essential. The critical role of electroactive biofilm in MFC performance has been repeatedly reported^50,54^. It was demonstrated that the biofilm formation at the highest external resistance is much more uniform compared to lower external resistance which facilitated the nutrient diffusion into it and accelerated release of electrons. In addition, the possibility of biofilm detachment in the uniform biofilm is less than non-uniform one. These were the main reasons that microbial enrichment was implemented under open circuit conditions. The evolution of OCP measuring during microbial enrichment is shown in Fig. 5. During the MFC operation, the volume of anolyte decreased due to microbial activity, substrate consumption and anolyte evaporation. Therefore, to prevent the reduction of cell’s potential, the anolyte was refilled with the fresh substrate. The injection of the fresh medium to refill the anolyte shown by arrows enhanced the OCP of the MFC remarkably.

The dynamic response of the system as the variation of system potential to the refreshment of the consumed substrate was visible as long as microbial inoculation continued during OCP evolution. A potential cycle during the evolution of MFC potential can be divided into three phases: ascending phase, stationary phase, and descending phase. An increase in the stationary phase (i.e., horizontal section) of OCP evolution indicated the organic saturation of MFC’s anolyte. In other words, MFC’s bacteria have sufficient food for growth and achievement of redox reaction. The observation of the stable OCP after three stationary phases indicated that the biocatalyst had stabilized and microbial enrichment was successful^55^. The maximum OCPs of 1070 and 1012 mV were obtained for the MFCs cultured *Z. mobilis* in minimal and modified medium, respectively.

It was observed that changing to the closed circuit by applying an external resistance and then returning to open circuit conditions by removing the external resistance could enhance biofilm growth and increase OCP^50,56^. Figure 5B and C demonstrated that changing the open circuit to the closed-circuit conditions by exerting 5.5 MΩ increased OCP to 1070 mV (more than 37%). This was done for both MFCs and a significant increase in the OCPs was observed. The controlling electrons flow in a circuit by an external resistance highly affects the growth of biofilm^57^. In other words, applying an external resistance catalyzes the electron generation of the new formed biofilm and accelerates its growth. This plays a critical role during the inoculation of the MFC^58^. Mclean. et al. demonstrated the anode potential correlated with biofilm development. When an external resistance of 1 MΩ was applied, the growth of *shewanella oneidensis* MR-1 increased and a thick biofilm on the graphite anode was obtained, whereas only a thin biofilm formed on the anode with an external resistance of 100 Ω. As a result, the anode potential was enhanced by the development of biofilm^59^.

Another interesting point in Fig. 5 is the significant value of OCP of the MFC (1070 mV) which can be attributed to the role of the metal-based electrode. The use of nickel anode in the MFC on the one hand and the presence of inorganic compounds in the medium (such as KH_2_PO_4_, K_2_HPO_4_, (NH_4_)_2_SO_4_, NaCl, etc.) establish a hybrid system and the MFC can also operate as a Nickel-carbon battery. This was proved in the previously published study in which the utilization of different metal-based electrodes increased the OCP of the MFC even higher than theoretical values^60^.

### The effect of external resistance

**Figure 6:**
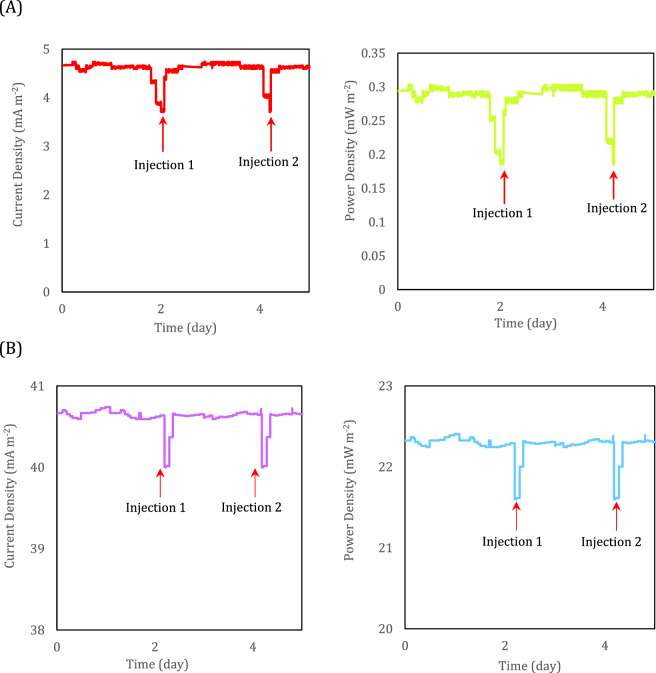
The evolution of current and power densities of (**A**) *Z. mobilis* + medium and (**B**) *Z. mobilis* + medium + CuCl_2_ (modified medium) at 15 kΩ external resistance.

Regarding the main objective of this study to demonstrate that the suggested procedure of metabolic engineering could improve the bioelectricity of *Z. mobilis* and produce much more electrons instead of ethanol, the effect of applying different external resistance should be investigated. For all electrogenesis microorganisms, the electron is extracted due to the conversion of NADH to NAD^+^^49^. Concerning the EET of *Z. mobilis*, as mentioned previously, the strategy of metabolic engineering should affect alcohol dehydrogenase of the fermentation pathway and prevent consumption of electron by converting NADH to NAD^+^ and producing ethanol and hence, the accumulated electron can be redirected toward generation of electricity. To show this variation in the electron production of electrogens, the MFCs were polarized by applying different external resistances. Figure 6 shows the current and power densities evolution of the MFCs fed with *Z. mobilis* with minimal and modified mediums. The gradual depletion of organic content in MFCs decreased the rate of electron production. As soon as microorganisms were fed with fresh nutrients the rate of electron production increased. For both MFCs, an injection of fresh medium, increased current and power densities abruptly. This increase was shown in Fig. 6 by arrows. The maximum current density and power density for *Z. mobilis* cultured in the minimal medium at 15 kΩ resistance were 4.6 mA m^−2^ and 0.29 mW m^−2^, respectively. Meanwhile, for the modified medium (namely *Z. mobilis* + medium + CuCl_2_), these values at the same external resistance were 40.00 mA m^−2^ and 21.60 mW m^−2^, respectively. This shows that the modification in the bacteria medium increased the power density by more than 77-folds.

**Figure 7:**
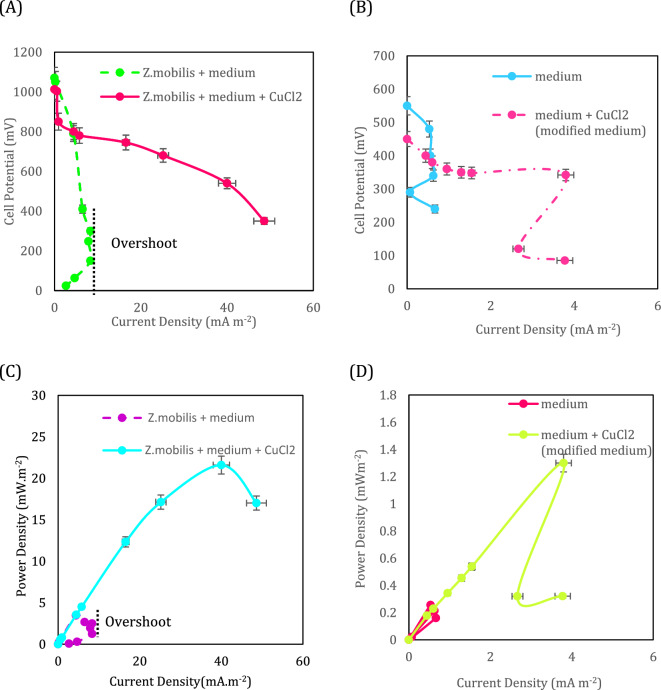
The polarization curves of the MFCs fed with the minimal and modified mediums (**A**) in presence of *Z. mobilis* (**B**) in absence of *Z. mobilis.* The power density curves of the MFCs fed with the minimal and modified mediums (**C**) in presence of *Z. mobilis* (**D**) in absence of *Z. mobilis.* The error bars represent the variation of current and power densities among repeated experiments.

Regarding the gradual reduction in the organic content of the MFCs, the polarization and power density should be irrespective of substrate concentration. As a result, by applying each external resistance, the refreshment of the medium was done and after 180 h, the maximum value of the cell potential was recorded. The range of the external resistances to polarize the cell was 10 kΩ to 5.5 MΩ. To assess the MFC’s over-potentials or the galvanic losses of MFCs, the polarization and power density curves are useful tools. Considering the overpotentials of an MFC including activation, ohmic and concentration overpotentials, the variations in each of them can be observed in separate sections of the polarization curves. The activation overpotential is the first section of the polarization curve describing the loss due to the metabolic reactions in extracting electrons and the required activation energy to run the redox reactions in both cathode and anode electrodes. The ohmic overpotential as a linear potential drop (remarked in the second section) represents the loss of electron transfer in the anolyte and the electrical connection. Finally, the concentration overpotential describes the limitation of the mass transfer of species during the redox reactions and through the proton exchange membrane as well^61^. The polarization and power density curves for the MFCs are shown in Fig. 7.

The modification of the bacteria medium increased the maximum current and power densities of the MFC fed with *Z. mobilis* by more than 5.8-folds and six-folds compared to the minimal medium, respectively (Fig. 7A and C). In addition, this modification played a critical role in decreasing the ohmic overpotential. As can be seen, the slope of the middle section of the polarization curve representing the ohmic overpotential for *Z. mobilis* + medium + CuCl_2_ (modified medium) decreased significantly. Therefore, the produced electrons in the anolyte of the modified medium had the least loss to reach the electron acceptor. The first speculation might attribute this matter to the enhancement of anolyte conductivity due to the CuCl_2_ presence but the polarization curve of the MFCs fed with the minimal and modified mediums without the bacteria presence revealed that the presence of this compound produced a maximum of 3.79 mA m^−2^ and 1.29 mW m^−2^ which were a small proportion of total current and power densities of the cell, respectively (Fig. 7B and D). Therefore, the electron production of *Z. mobilis* in the MFC with a modified medium brought about such enhancement in the current and power densities. The electron production in the MFC was chiefly achieved by the presence of this bacteria and the modification of the medium, in addition, to improving the conductivity of the anolyte, exceedingly decreased the activation loss of bacteria during the electron’s extraction in the metabolic reactions.

One of the primary motivations behind the current study is to demonstrate the importance of metabolic engineering in enhancing the performance of MFCs enriched with bacteria that do not possess a remarkable tendency for electron generation. *Z. mobilis* is a bacterium known for its high ethanol production rather than electron generation. Considering previously published studies^34^, only one study has investigated the electrochemical performance of *Z. mobilis* in MFCs. The power density obtained from the cell inoculated with this species was approximately 2.0 (mW/m^2^), which was quite low. However, by enhancing the electron production pathways in *Z. mobilis* through metabolic engineering, the power generation increased to 21.6 (mW/m^2^). This accomplishment highlights the importance of metabolic engineering in strengthening electrogenesis pathways, a strategy with the potential for broader application to various microorganisms. It can significantly accelerate the commercialization of Microbial Fuel Cells (MFCs), particularly when the limited performance of introduced microorganisms poses a significant hurdle. Consequently, future research endeavors should consider adopting this approach or investigating alternative electrogenic species.

The overshoot phenomenon as a result of sudden electron depletion which decreases the current density was observed for *Z. mobilis* with the minimal medium (Fig. 7A, green dashed curve). In this situation, at low external resistance, the electrons suddenly passed the circuit and this led to an excessive demand for electrons which cannot be compensated by the metabolic reactions of bacteria. Consequently, the current density of the MFC dropped. Insufficient mass transport of redox species particularly the anodic reactions play a remarkable role in the occurrence of the overshoot phenomenon^62^. The modification of the medium could prevent the occurrence of the overshoot phenomenon by deactivation of alcohol dehydrogenase and provide sufficient NADH for electron extraction during an abrupt depletion of electrons at low external resistances. Based on the data of the system, the coulombic efficiencies of 0.511 and 0.63 for the minimal and modified medium, respectively, were calculated. Thus, the result demonstrates about 23% improvement in the coulombic efficiency for the modified medium compared to the minimal.

### Biofilm morphology

**Figure 8:**
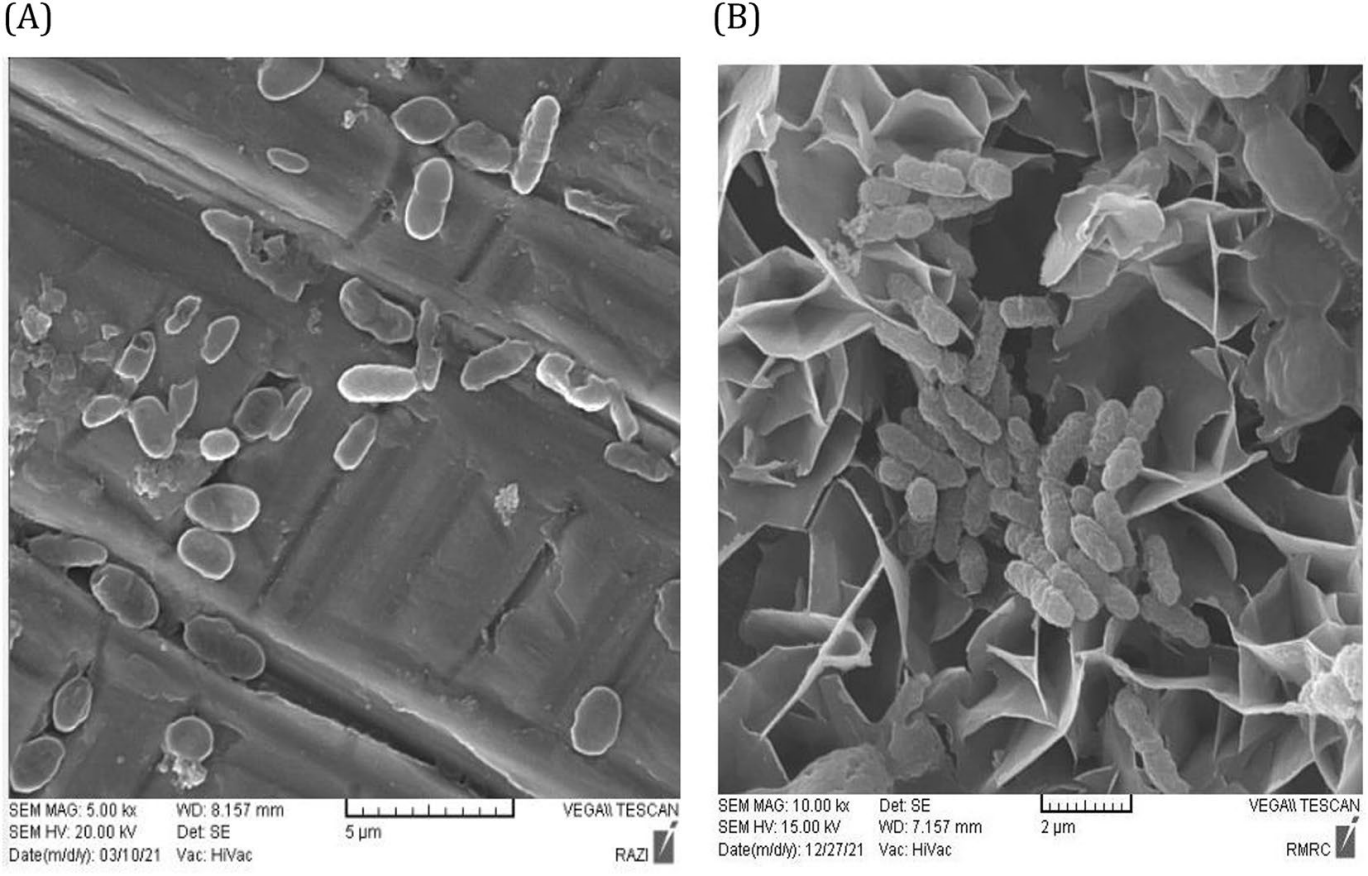
Field emission scanning electron microscopy (FESEM) of *Z. mobilis* biofilm in (**A**) minimal and (**B**) modified mediums at 5000 and 10,000 magnifications.

An enhancement of electron production causes the accumulation of bacteria on the surface of the electrodes. This accumulation can be observed as a biofilm formed on the anode surface of MFCs playing a critical role as a final electron acceptor^63^. To confirm the accumulation of biomass on the anode surface of the MFCs inoculated with *Z. mobilis* in both minimal and modified medium, the images of field emission scanning electron microscopy (FESEM) were analyzed and the morphology of these MFCs anodes is shown in Fig. 8. The modification of bacteria medium increased the tendency of *Z. mobilis* to electron production and the higher aggregation of them on the anode surface of MFC with the modified medium is crystal-clear evidence for this claim (Fig. 8B). As the enhancement of biofilm density improves the power generation of cells^50^, the higher bioelectricity generation of the *Z. mobilis* in the modified medium can be attributed to the formation of the denser biofilm on the anode surface compared to the MFC’s anode fed with the minimal medium.

## Conclusion

**Figure 9:**
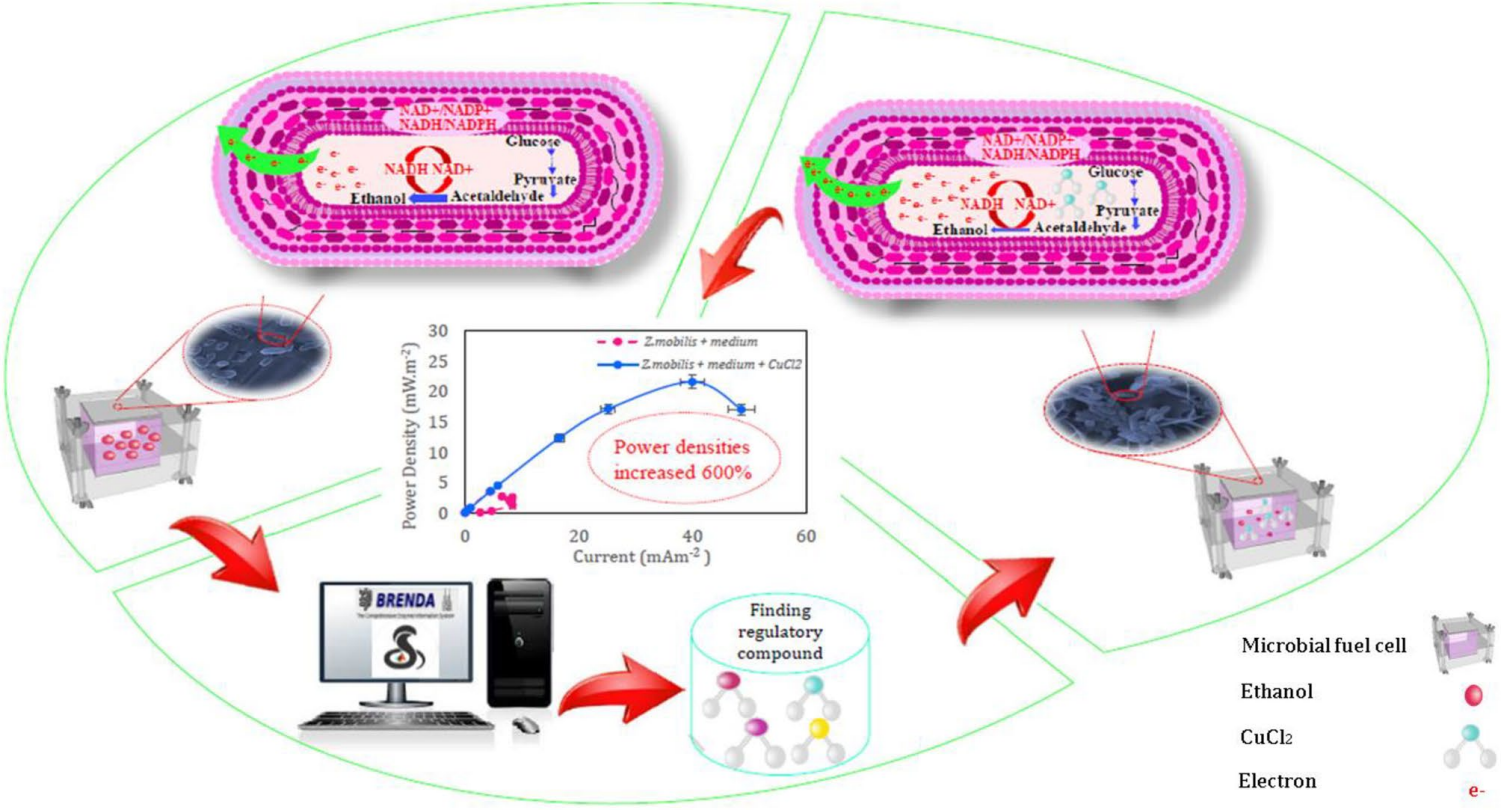
The graphical abstract of the present study demonstrates how metabolic engineering reinforces electron generation pathways to enhance MFC performance.

To being with, a compreheansive analysis of the metabolic pathways involved in bioelectricity generation is conducted. This analysis helps identify key bottleneck genes that effect the efficiency of these pathways. Bottleneck genes are those that have a significant impact on the overall metabolic flux and can potentially be targeted for improvement. LAMOS and BRENDA are compoutational tools used for the initial screening of existing compounds to identify potential candidates for switching metabolism and enhancing NADH production by analyzing the structure activity of compounds, these tools can predict their potential to modulate metabolic pathways and increase power density generation(Fig. 9.). The metabolic engineering of *Zymomonas mobilis* to modify its growth medium and enhance bioelectricity production has been successfully accomplished. This study has provided evidence that CuCl_2_ serves as an effective regulator, influencing the activity of alcohol dehydrogenase in the pyruvate-to-ethanol pathway. By promoting the conversion of NADH to NAD^+^, CuCl_2_ increases the rate of electron production. Moreover, the modification of the growth medium has significantly reduced the overpotentials observed in microbial fuel cells (MFCs), thereby facilitating the efficient transfer of generated electrons.

The medium modification has also proven beneficial in preventing the occurrence of the overshoot phenomenon. By deactivating alcohol dehydrogenase and ensuring sufficient NADH availability, the modified medium enables the extraction of electrons even under conditions of sudden electron depletion at low external resistances. Furthermore, the accumulation of *Z. mobilis* biofilm on the anode surface further supports the role of CuCl_2_ as a regulatory enzyme in enhancing MFC performance.

In summary, this study demonstrates the successful metabolic engineering of *Z. mobilis* and the efficacy of CuCl_2_ as a regulator, leading to improvements in bioelectricity production. The modification of the growth medium has shown promising results in reducing overpotentials, preventing overshoot, and promoting biofilm formation, ultimately enhancing the overall performance of MFCs.

## Acknowlegements

The authors wish to acknowledge Tarbiat Modares University for its financial support of this research.

### Supplementary Information

Supplementary Information.

## Data Availability

All data generated or analysed during this study are included in this published article and in supplementary file 1.
