# Supplementary material for: Metabolic regulation boosts bioelectricity generation in Zymomonas mobilis microbial fuel cell, surpassing ethanol production
Source: Sci Rep. 2023 Nov 24;13:20673. doi: 10.1038/s41598-023-47846-7 (PMC10673858; doi:10.1038/s41598-023-47846-7)
Supplement: Supplementary file 1 — Supplementary Information. [file 41598_2023_47846_MOESM1_ESM.docx]

**Metabolic regulation boosts bioelectricity generation in *Zymomonas mobilis* microbial fuel cell, surpassing ethanol production**

Hananeh Ahmadpanah^1^, Ehsan Motamedian^1^, Mohammad Mahdi Mardanpour^2^

*^1^ Department of Biotechnology, Faculty of Chemical Engineering, Tarbiat Modares University, P.O. Box 14115‑143, Tehran, Iran.*

*^2^ Department of Bioengineering, Faculty of Engineering, McGill University, Montreal, Canada*

**1. The influential reactions to improve NADH production.**

**Table S1.** Predicted reactions for up-regulation to improve NADH production.

| Reaction name | Reaction Description | Formula | Subsystem | Gene | EC Number |
| --- | --- | --- | --- | --- | --- |
| ALDD2y | aldehyde dehydrogenase | acald[c] + h_2_o[c] + nadp[c] -> ac[c] + 2h[c] + nadph[c] | Glycolysis  /Gluconeogenesis | - | 1.2.1.4 |
| G6PDH1r_b | beta-D-Glucose-6-phosphate | g6p[c] + nadp[c] <=> 6pgl[c] + h[c] + nadph[c] | pentose phosphate | Zwf | ALDD2y |
| G6PDH2r_f | beta-D-Glucose-6-phosphate | g6p[c] + nad[c] <=> 6pgl[c] + h[c] + nadh[c] | pentose phosphate | Zwf | 1.1.1.49 |
| Htex_b | proton transport via diffusion | h[e] <=> h[c] | - | - | - |
| H2Otex_f | H2O transport via diffusion | h_2_o[e] <=> h_2_o[c] | - | - | - |

**Table S2.** Predicted reactions for down-regulation to improve NADH production.

| Reaction name | Reaction Description | Formula | Subsystem | Gene | EC Number |
| --- | --- | --- | --- | --- | --- |
| ALCD2x_b | alcohol dehydrogenase | etoh[c] + nad[c] <=> acald[c] + h[c] + nadh[c] | Glycolysis  /Gluconeogenesis | AdhP | 1.1.1.1 |
| G6PDH1r_f | beta-D-Glucose-6-phosphate | g6p[c] + nadp[c] <=> 6pgl[c] + h[c] + nadph[c] | pentose phosphate | Zwf | ALDD2y |
| FORtex_b | formate transport via diffusion | for[e] <=> for[c] | - | - | - |
| H2Otex_b | H2O transport via diffusion | h2o[e] <=> h2o[c] | - | - | - |
| SO4tex_b | sulfate transport via diffusion | so4[e] <=> so4[c] | - | - | - |

**2. Ethanol analysis of cultured *Z. mobilis* in its medium without regulatory compounds**

**Fig. S1.** The assessment of the ethanol production of *Z. mobilis* over time in cultured *Z. mobilis* in its medium without regulatory compounds.

**3. Ferrozine analysis of cultured *Z. mobilis* in its medium without regulatory compounds**

**Fig. S2.** The assessment of the electron production of *Z.mobilis* over time in cultured *Z.mobilis* in its medium without regulatory compounds using measurement of the evolution of Fe (III) reduction.
